# Chronic Regulation of miR-124-3p in the Perilesional Cortex after Experimental and Human TBI

**DOI:** 10.3390/ijms21072418

**Published:** 2020-03-31

**Authors:** Niina Vuokila, Eleonora Aronica, Anatoly Korotkov, Erwin Alexander van Vliet, Salma Nuzhat, Noora Puhakka, Asla Pitkänen

**Affiliations:** 1 A. I. Virtanen Institute for Molecular Sciences, University of Eastern Finland, P.O. Box 1627, FI-70211 Kuopio, Finland; niina.vuokila@uef.fi (N.V.); salnu@student.uef.fi (S.N.); asla.pitkanen@uef.fi (A.P.); 2 Department of (Neuro)pathology, Amsterdam UMC, University of Amsterdam, Meibergdreef 9, 1105 AZ Amsterdam, The Netherlands; e.aronica@amsterdamumc.nl (E.A.); a.korotkov@amc.uva.nl (A.K.); e.a.vanVliet@uva.nl (E.A.v.V.); 3 Stichting Epilepsie Instellingen Nederland (SEIN), 0397 Heemstede, The Netherlands; 4 Swammerdam Institute for Life Sciences, Center for Neuroscience, University of Amsterdam, Science Park 904, P.O. Box 94246, 1090 GE Amsterdam, The Netherlands

**Keywords:** bioinformatics, biomarker, epileptogenesis, microRNA, miR-124-3p, traumatic brain injury

## Abstract

Traumatic brain injury (TBI) dysregulates microRNAs, which are the master regulators of gene expression. Here we investigated the changes in a brain-enriched miR-124-3p, which is known to associate with major post-injury pathologies, such as neuroinflammation. RT-qPCR of the rat tissue sampled at 7 d and 3 months in the perilesional cortex adjacent to the necrotic lesion core (aPeCx) revealed downregulation of miR-124-3p at 7 d (fold-change (FC) 0.13, *p* < 0.05 compared with control) and 3 months (FC 0.40, *p* < 0.05) post-TBI. In situ hybridization confirmed the downregulation of miR-124-3p at 7 d and 3 months post-TBI in the aPeCx (both *p* < 0.01). RT-qPCR confirmed the upregulation of the miR-124-3p target *Stat3* in the aPeCx at 7 d post-TBI (7-fold, *p* < 0.05). mRNA-Seq revealed 312 downregulated and 311 upregulated miR-124 targets (*p* < 0.05). To investigate whether experimental findings translated to humans, we performed in situ hybridization of miR-124-3p in temporal lobe autopsy samples of TBI patients. Our data revealed downregulation of miR-124-3p in individual neurons of cortical layer III. These findings indicate a persistent downregulation of miR-124-3p in the perilesional cortex that might contribute to post-injury neurodegeneration and inflammation.

## 1. Introduction

Traumatic brain injury (TBI) is a leading cause of death and disability, affecting more than 50 million people worldwide each year [1]. After the primary impact, several secondary molecular and cellular pathologies develop, including cellular reorganization, immune responses, progressive neuronal loss, and glial scarring [2,3]. Some cellular changes, such as neurogenesis or axonal/dendritic plasticity, support recovery [4,5]. There are currently no treatments to halt the progression of post-TBI pathologies or facilitate the recovery-enhancing molecular and cellular changes. Thus, there is a major unmet medical need to identify targets for intervention with small molecules or biologics to improve the prognosis of TBI.

MicroRNAs (miRNAs) are small (18–23 nucleotides) non-coding RNAs [6,7] that suppress gene expression by binding to the 3′ untranslated region (3′UTR) of messenger-RNA, thus preventing its translation [7]. The miRNA-target(s) interaction depends on the miRNA seed region sequence [8]. Thus, possible targets can be predicted on the basis of the mRNA 3′UTR sequence. Some miRNAs might have hundreds of possible targets [8]. A change in miRNA expression can therefore lead to remarkable changes in cellular function. miRNA-based treatments show potential as disease-modifying network therapies after experimental TBI [9,10,11].

Despite an increasing number of reports describing alterations in the regulation of miRNAs after TBI, our understanding of the regulation of the miRNome and its cellular localization after TBI is still in its infancy [12,13]. TBI regulates brain miRNA expression acutely [14,15,16,17], and some miRNAs remain regulated chronically—months to years after TBI [18,19]. Dysregulated miRNA levels are reported in the cortex [9,14,20,21,22], hippocampus [15,16,18,19,23,24,25,26,27], and plasma [28,29,30]. Only a few studies, however, have addressed miRNA expression in patients with TBI [13,19,23,28], highlighting the gap between preclinical and clinical studies. Our previous study showed that TBI results in miR-124-3p downregulation in both rat and human dentate gyrus, making this miRNA an interesting subject for further study.

miR-124 is the most abundant microRNA in the brain, accounting for 25–48% of all brain miRNA pool [31]. It is crucial for neuronal development [32,33], and immune responses [34]. miR-124-3p drive cells towards neuronal identity [35,36], and regulates gene expression by promoting neuron-specific splicing [37]. Our previous study on post-TBI downregulation of miR-124-3p in the dentate gyrus suggested a link between miR-124-3p and post-injury hippocampal pathologies such as epileptogenesis and memory decline [19]. To explore the role of miR-124-3p in the progression of cortical damage, we investigated the post-TBI expression pattern of miR-124-3p in the perilesional cortex in experimental and human TBI. To investigate the miR-124-3p–controlled reactome, we conducted in silico analysis on the miRNA-target binding regions.

## 2. Results

### 2.1. Animals

#### 2.1.1. Mortality

Acute mortality within 48 h post-TBI was 0% in Cohort 1, 25% (3/12) in Cohort 2, and 17% (3/18) in Cohort 3. In Cohorts 1 and 2, one control rat died during the surgery.

#### 2.1.2. Time in Apnea

The mean post-impact apnea was 25 s for Cohort 1, 35 s for Cohort 2, and 21 s for Cohort 3.

#### 2.1.3. Acute Post-Impact Seizure-Like Behaviors

Post-impact seizure-like behavior was observed in 13% of animals in Cohort 1 (mean event duration: 25 s), 50% of animals in Cohort 2 (mean event duration: 35 s), and 22% of animals in Cohort 3 (mean event duration: 21 s).

### 2.2. RT-qPCR Analysis Indicated Downregulation of miR-124-3p in the Perilesional Cortex (Cohort 1)

**Figure 1:**
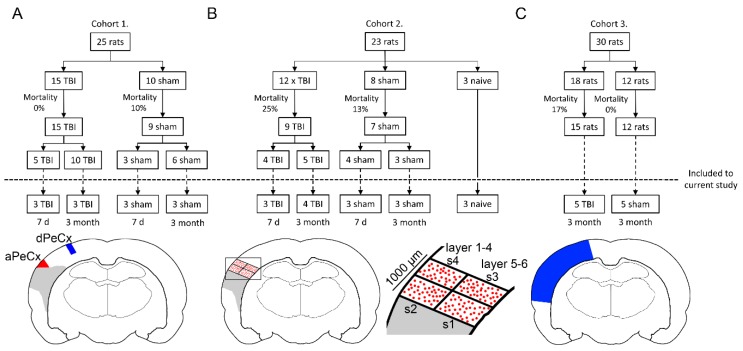
Study design and tissue sampling. (**A**) Cohort 1: a total of 25 animals underwent either traumatic brain injury (TBI; *n* = 15) or sham (*n* = 10) operation. Of the surviving 24 animals, 8 (5 TBI, 3 sham) were killed at 7 d post-TBI, and the rest (10 TBI, 6 sham) at 3 months post-TBI. Six TBI and 6 sham animals were randomly chosen for RT-qPCR analysis. Two samples were dissected for analysis: the perilesional cortex adjacent to the lesion core (aPeCx, red) and the somatosensory cortex distal to the lesion (dPeCx, blue). (**B**) Cohort 2: a total of 23 animals underwent either TBI (*n* = 12) or sham (*n* = 8) operation. From the surviving 16 animals 8 (4 TBI, 4 sham) were killed at 7 d post-TBI, and the remaining animals (5 TBI, 3 sham) at 3 months post-TBI. Altogether 7 TBI and 6 sham rats were used for miR-124-3p in situ hybridization. The remaining 3 animals did not undergo any surgical procedures (naïve controls). Analysis of miR-124-3p expression in in situ-processed sections was conducted in individual cells (red dots) within a 1000-µm thick sector adjacent to the lesion core. A corresponding area was analyzed in the control samples. (**C**) Cohort 3: a total of 30 animals underwent either TBI (*n* = 18) or sham (*n* = 12) operation. At 3 months post-TBI, animals were killed and the brain was cut into 2-mm thick coronal slices (bregma levels from −2.2 to −6.2) and tissue from the perilesional cortex (blue area in A) was sampled for RNA-sequencing.

**Figure 2:**
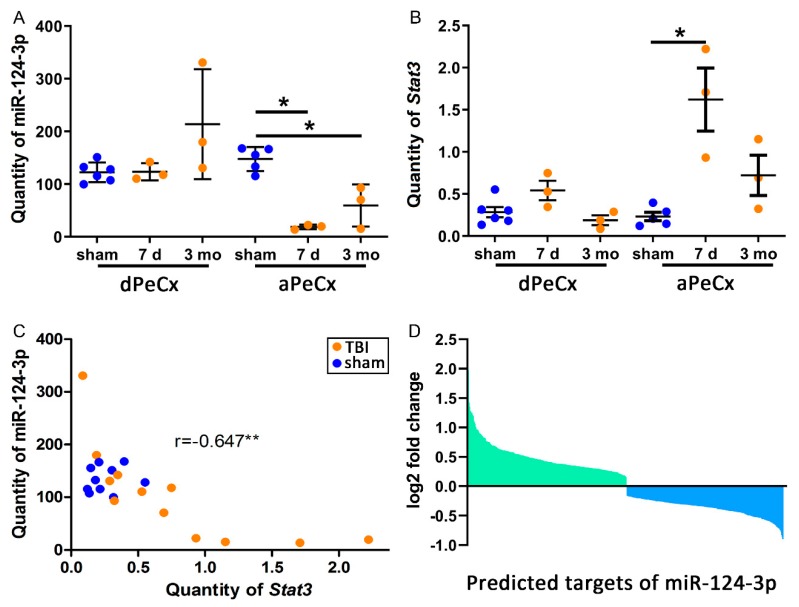
TBI downregulates miR-124-3p and upregulates *Stat3* in the perilesional cortex. (**A**) A RT-qPCR analysis of laser-capture microdissected cortex indicated a downregulation of miR-124-3p in the perilesional cortex adjacent to the lesion core (aPeCx, FC 0.13 at 7 d, FC 0.40 at 3 months; both *p* < 0.05) but not in the more distal perilesional cortex (dPeCx, approximately 600 µm more dorsal) at both 7 d and 3 months post-TBI. (**B**) Expression of *Stat3*, one of the targets of miR-124-3p, was upregulated in aPeCx at 7 d post-TBI (*p* < 0.05, FC = 6.97) and tended to be upregulated at 3 months post-TBI (*p* > 0.05, FC = 3.11). (**C**) A correlation analysis revealed that the higher the miR-124-3p levels, the lower the *Stat3* levels (r = −0.647, *p* < 0.01). (**D**) RNA sequencing revealed 312 upregulated and 311 downregulated targets of miR-124-3p. The gene set enrichment analysis (GSEA) of these targets indicated that only the downregulated targets were enriched in the dataset (ES = −0.33, FDR < 0.01). dPeCx, distal part of cortex; d, day; ES, enrichment score; FC, fold change; FDR, false discovery rate; LCM, Laser capture microdissection; mo, month; aPeCx, perilesional cortex; RT-qPCR, quantitative reverse transcriptase polymerase chain reaction. Statistical significance: *p < 0.05, **p < 0.01 (Mann–Whitney U).

To investigate the spatiotemporal variation in miR-124-3p expression after TBI relative to the lesion location, we used RT-qPCR to evaluate miR-124-3p expression in the perilesional cortex adjacent to the necrotic lesion core (aPeCx) and somatosensory cortex distal to the lesion (dPeCx; see Figure 1A). Tissue was sampled at 7 d and 3 months post-TBI (see Figure 1). RT-qPCR analysis revealed no difference between TBI and sham-operated control animals in the dPeCx samples at either time-point (Figure 2A). Analysis of aPeCx showed that miR-124-3p was downregulated at both 7 d (fold-change 0.13, *p* < 0.05) and 3 months post-TBI (fold-change 0.40, *p* < 0.05, Figure 2A) compared with sham-operated controls. The miR-123-3p levels were not significantly different between the 7-d and 3-month post-TBI groups.

### 2.3. TBI Disrupts the Cortical Expression Pattern of miR-124-3p in Rats (Cohort 2)

**Figure 3:**
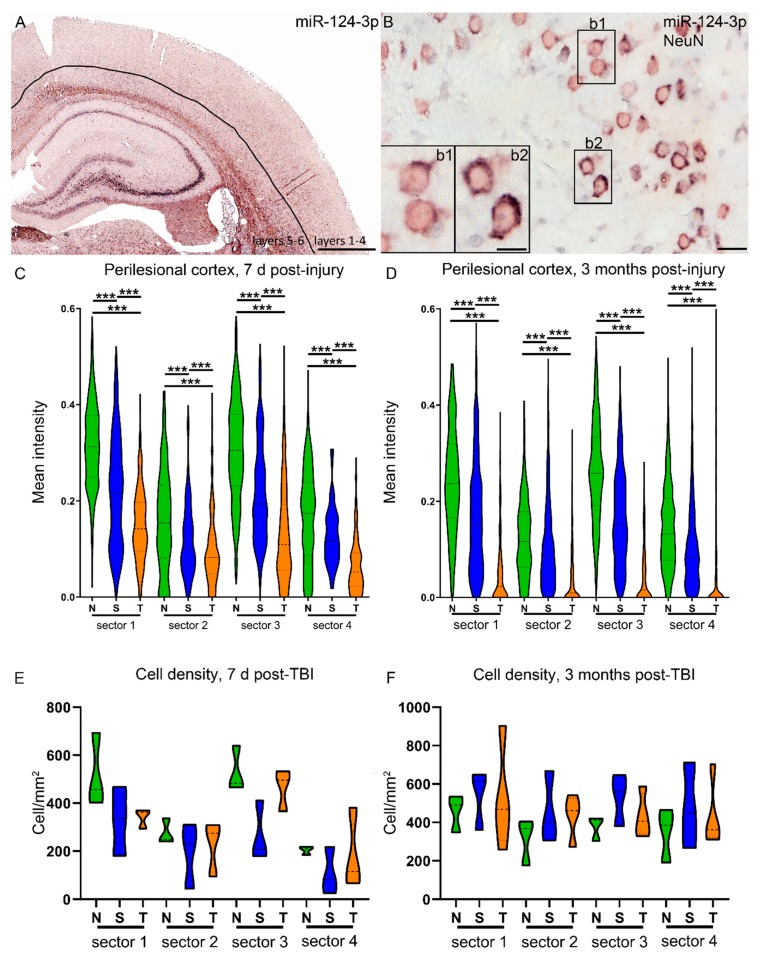
Perilesional expression of miR-124-3p is downregulated by lateral fluid-percussion injury as well as by craniectomy. (**A**) In situ hybridization was used to detect the distribution and intensity of cellular expression of miR-124-3p in different layers of the cortex. (**B**) Double-labeling with NeuN revealed that the expression level of miR-124-3p varied between the neurons in the perilesional cortex (inserts b1, b2). Our analysis focused on a 1-mm wide area of the dorsal perilesional cortex, which was further divided into four sectors (see Figure 1). The intensity of in situ hybridization signal was measured in the individual neurons within each sector (mean number of cells counted: 7 d, naïve 123; sham 89; TBI 94; 3 months, naïve 125; sham 151; TBI 63). (**C**,**D**) Naïve animals (N) showed a clear expression pattern at both 7 d and 3 months post-TBI, with a higher level of miR-124-3p expression in the deeper (s1, s3) than superficial layers (s2, s4). The proximal and distal sectors had similar expression levels (s1 vs. s3; s2 vs. s4). In sham-operated experimental controls (S) with a craniotomy, the overall pattern of miR-124-3p expression was preserved. At both time-points and within each of the four sectors, however, the miR-124-3p levels were downregulated compared with those in naïve animals (*p* < 0.001). In rats with TBI (T), the miR-124-3p expression levels were even lower than those in sham-operated animals (*p* < 0.001) in all sectors and at both time-points. (**E**,**F**) The cell density in the sectors did not differ between time-points or groups. N, naïve; S, sham; s1–s4, sector as described in Figure 1; T/TBI, traumatic brain injury. Statistical significance: *** *p* < 0.001 (Mann–Whitney U). Scale bars: A 1000 µm, B 20 µm, insert in B 10 µm.

Next, we confirmed that the downregulation of miR-124-3p in the perilesional cortex was not related to the shift in the cellular composition; that is, neuronal cell loss or an increase in neuroinflammatory cells. Based on the in situ hybridization coupled with double labeling with cellular markers for astrocytes (GFAP), microglia (Iba1), and neurons (NeuN), we know that miR-124-3p is neuronal miRNA (Supplementary Figure S1) In situ hybridization revealed a higher expression of miR-124-3p in the deeper cortical layers than in the superficial cortical layers (Figure 3A). Moreover, miR-124-3p in situ hybridization coupled with NeuN immunohistochemistry indicated that the neuronal miR-124-3p expression varied in the perilesional cortex (inserts in Figure 3B). To further investigate whether TBI induced a change in the (a) number of miR-124-3p–positive cells, (b) distribution of the miR-124-3p positive cells, or (c) expression of miR-124-3p in individual cells, we performed a quantitative analysis of in situ hybridization preparations of naïve, sham, and TBI animals. The intensity of the miR-124-3p signal in individual cells was quantified along a 1-mm wide sector divided into four areas, starting from the dorsal edge of the lesion, as indicated in Figure 1B. In naïve animals (green plots in Figure 3C,D), miR-124-3p expression was higher (7 d: naïve deeper vs. superficial, FC 1.9; sham FC 1.8; TBI FC 1.6; 3 mo: naïve deeper vs. superficial, FC 1.8; sham FC 1.9; TBI FC 1.1, all *p* < 0.01) in the deep layers than in the superficial layers. Moreover, in the perilesional cortical sample, miR-124-3p expression levels were comparable in the proximal and distal sectors. In sham-operated experimental controls with a craniotomy, the overall pattern of miR-124-3p expression was somewhat preserved, with deeper layers having a higher level of miR-124-3p than superficial layers (blue plots Figure 3C,D). In the TBI rats, the expression pattern was remarkably changed at both 7 d and 3 months post-TBI (orange plots Figure 3C,D). The TBI animals had lower miR-124-3p expression levels than naive or sham-operated animals (7 d: 47% and 66%, respectively; both *p* < 0.01; 3 months: 24% and 35%, respectively; both *p* < 0.01). Interestingly, sham-operated experimental controls also had lower expression levels than naive animals (7 d: 71%, *p* < 0.01; 3 months: 69%, *p* < 0.01). Importantly, the density of miR-124-3p–positive cells within the four sectors analyzed did not differ between groups (Figure 3E,F).

### 2.4. TBI Causes Downregulation of miR-124-3p in the Human Cortex

As miR-124-3p is broadly conserved among species, we next investigated whether the findings in a rat model of TBI with a lesion epicenter in the auditory association cortex could be validated in human brain tissue samples.

**Figure 4:**
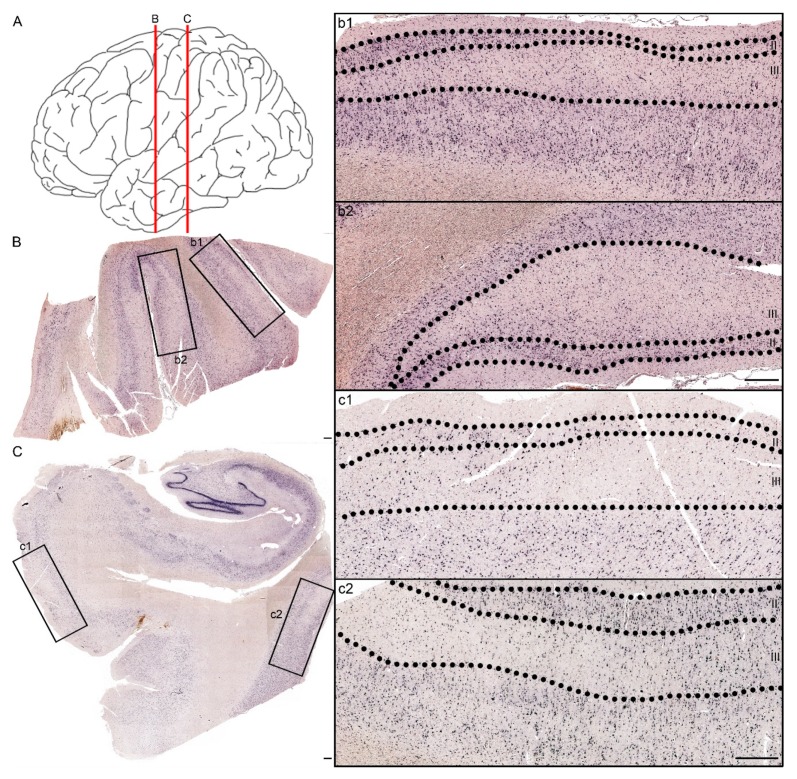
Patchy loss of miR-124-3p expression in the temporal cortex of a patient with severe TBI 5 days earlier. (**A**) Red lines indicate the coronal levels of sections illustrated in panels B,C. (**B**) miR-124-3p in situ hybridization from a section of the lateral temporal cortex. Note the neuronal loss in higher magnification images, particularly in layer III (b1, b2 correspond to boxed areas in panel B). (**C**) Hippocampus, entorhinal cortex (higher magnification in panel c1), and more lateral temporal cortex (c2). Note the preservation of the miR-124-3p hybridization signal in layer II and its loss in layer III. Scale bar: 500 µm.

Human temporal lobe tissue was available from six patients, three of whom had experienced TBI (Table 1). Ten sections available from these patients were used for miR-124-3p in situ hybridization. Analysis of cortical sections showed that all of the TBI patients had patchy neuronal loss as well as reduced miR-124-3p signal, which was most prominent in layer III (Figure 4).

**Figure 5:**
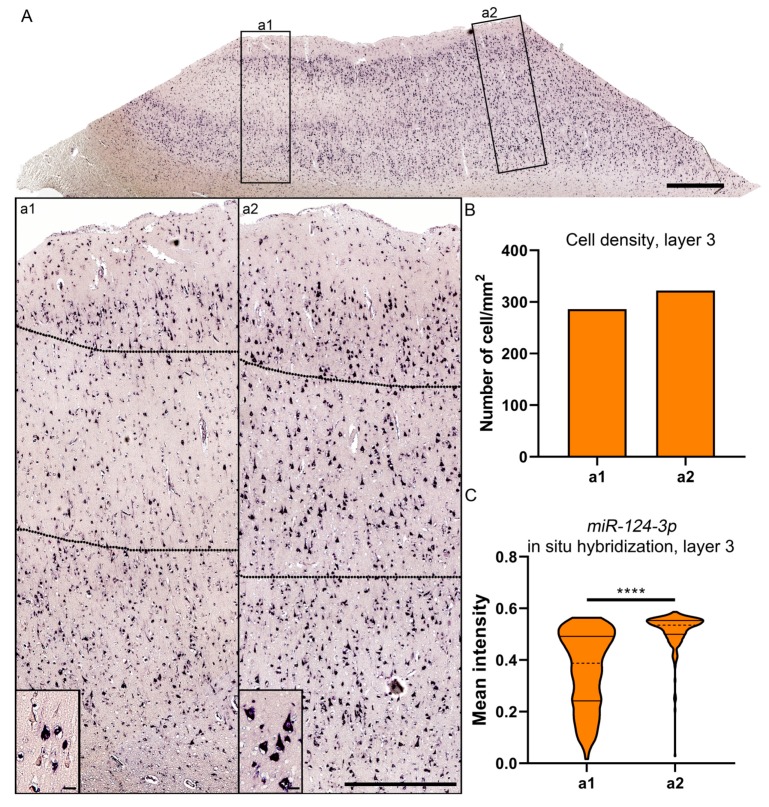
Expression of miR-124-3p in layer III cells of the lesioned cortex in a patient with severe TBI 5 days earlier. (**A**) Expression of miR-124-3p inside (a1) and outside (a2) the area with a visible loss of miR-124-3p. (**B**) Cellular density was comparable in the investigated areas. (**C**) Violin plots showing the greater variability in the distribution of the mean miR-124-3p signal intensity inside (a1) than outside (a2) the patchy neuronal loss region. The mean intensity of neuronal miR-124-3p in situ hybridization signal was lower inside than outside the lesion area (*p* < 0.01). Statistical significance: **** *p* < 0.001. Scale bars: A 1000 µm; a1 and a2 1000 µm; insert in a1 and a2 20 µm.

To further investigate the cellular expression of miR-124-3p, we outlined two 1000-µm wide sectors perpendicular to the pial surface, one inside and another outside of the patchy area in layer III, as shown in Figure 5. We detected no difference in the cellular density between the areas (Figure 5B). The intensity of the in situ hybridization signal, however, appeared lower inside than outside the patchy area (Figure 5C). We then measured the intensity of the miR-124-3p in situ hybridization signal in individual cells. Inside the patchy area, the miR-124-3p signal intensity varied widely between the positive cells (insert in Figure 5a1). Outside the patchy area, intracellular expression of miR-124-3p was more consistent (insert in Figure 5a2).

### 2.5. Bioinformatics Analysis Indicated post-TBI Regulation of miR-124-3p Targets in the Perilesional Cortex (Cohort 3)

Next, we assessed whether chronic downregulation of miR-124-3p associated with regulation of its target mRNAs in the rat cortex. TargetScan revealed 1547 predicted targets of miR-124-3p. Forty percent (623/1547) of the predicted targets of miR-124-3p were regulated after TBI. Our RNA-sequencing indicated an upregulation of 2583 and a downregulation of 2381 genes (*p* < 0.05) in the perilesional cortex at 3 months post-TBI. Of the 2583 upregulated genes, 20% (312) were miR-124-3p targets. Of the 2381 downregulated genes, 20% (311) were miR-124-3p targets. Despite a similar fold-change (Figure 2D) and number of regulated targets, the gene set enrichment analysis (GSEA) indicated enrichment of the downregulated targets in the dataset (effect size (ES) = −0.32, *p* < 0.01, false discovery rate (FDR) <0.01). Interestingly, when the minimum free energy between miR-124-3p and the top 10 targets from both ends of the GSEA enrichment plot were calculated using RNAhybrid (Supplementary Figure S2A,B), the downregulated targets had a lower minimum free energy (MFE, Supplementary Figure S2C), indicating a more stable bond between miR-124-3p and the target mRNA. The same analysis, however, showed that the optimal binding between the target and miRNA started in most cases from nucleotide 3-4 (5′). This was also case for *Stat3* (Supplementary Figure S2D), whose binding to miR-124-3p has been demonstrated multiple times [38,39,40]. Our PCR analysis indicated that the *Stat3* level did not vary in the dPeCx samples between sham and TBI animals (Figure 2B), while we found a 7-fold upregulation in the 7-d aPeCx samples (*p* < 0.05, Figure 2B). We found no significant difference between sham and 3 months post-TBI samples. Nevertheless, the miR-124-3p level correlated with the *Stat3* level (*r* = −0.647, *p* < 0.01, Figure 2C), suggesting that the increase in the *Stat3* level was associated with the miR-124-3p downregulation.

Next, we investigated the functions of the miR-124-3p–controlled molecular pathways. Upregulated and downregulated targets were mapped to known pathways using Reactome (20 pathways with most entities found, Table 2.). For upregulated targets, we found 45 subpathways (*p* < 0.05) clustered under 14 major pathways. The top three pathways with the most subpathways were the signal transduction (12/45, 27%), gene expression control (5/45, 11%), and programmed cell death (5/45, 11%). For downregulated targets, we found 55 subpathways (*p* < 0.05) clustered under 8 major pathways. The top three pathways with the most subpathways were the neuronal system-related (13/55, 24%), immune system (13/55, 24%), and signal transduction (11/55, 20%).

To further investigate the functional role of regulated miR-124-3p targets after TBI, we used ingenuity pathway analysis (IPA) analysis (Table 3.). The top 20 disease- or function-related pathways with the most upregulated targets were mainly categorized as nervous system development (10/20, 50%) or cell death and neurodegeneration (9/20, 45%; Table 3). Additionally, the top 20 disease- or function-related pathways with the most downregulated targets were mainly categorized as nervous system development (10/20, 50%) or neurodegeneration (7/20, 35%; Table 3). A more detailed description of the pathways within each main category is presented in Table 3.

## 3. Discussion

In the present study, we aimed to identify the molecular mechanisms underlying post-TBI cortical pathology. We hypothesized that TBI downregulates cortical neuron-enriched miR-124-3p, which regulates apoptosis, neuroinflammation, and cellular migration—all part of the post-TBI aftermath. We observed a long-lasting downregulation of miR-124-3p in the perilesional cortex in both experimental and human TBI. Our in silico analysis revealed a remarkable number of both upregulated and downregulated targets of miR-124-3p that function in a myriad of potentially disease-modified pathways after TBI.

### 3.1. TBI Causes Downregulation of miR-124-3p in the Perilesional Cortex

We previously demonstrated that miR-124 was downregulated in the dentate gyrus of rats with lateral FPI and in humans with TBI. The present study expanded these findings by revealing a robust downregulation of miR-124-3p in the perilesional cortex adjacent to the lesion, whereas in the more distal cortex, the miR-124-3p levels were comparable to that in sham-operated experimental controls. We also found that miR-124-3p was downregulated in human perilesional cortex, indicating the translatability of the animal data to the human condition. Recently, Miao and coworkers [21] reported miR-124-3p downregulation in the ipsilateral cerebral cortex in mice exposed to controlled cortical impact-induced TBI. Together, these data suggest that miR-124-3p downregulation is a common post-TBI molecular pathology that does not depend on the model or species investigated.

Many mature neuron-specific miRNAs have a relatively short half-life [41,42,43]. Moreover, in *Aplysia californica*, synaptic activity evoked by serotonin exposure downregulates miR-124 [43]. We previously reported that the precursors of hippocampal miR-124-3p are not affected by TBI [19]. This suggests that miR-124 downregulation is not caused by differential gene expression, but rather by dampening of the mature miR-124 processing; its degradation; or preventing its binding to targets, probes, and primers. Here we assessed a miRNA processing deficit as a possible cause of the miR-124-3p downregulation. We retrieved miRNA maturation-related molecular signature gene sets (GSEA) and compared them with our sequencing data (data not shown). The mRNA coding proteins related to miRNA maturation (based on the GSEA MsigDB [44]) were not changed in our dataset, except for Mrpl44 (Supplementary Table S1). Mrpl44 codes for the Mitochondrial Ribosomal Protein L44 and participates in pre-miRNA processing [36]; a change in this protein alone, however, is not proof of a miRNA maturation deficit.

While miRNA-mediated silencing of mRNAs has been studied extensively, the regulation of mature miRNAs has been little investigated. In plants, uridylation of the 3′ end of mature miRNA leads to its degradation [45], but studies in mammals are limited. Human cells contain three proteins that can degrade miRNAs, polynucleotide phosphorylase 1 [46], ribosomal RNA processing protein 41, and 5′-3′ exoribonuclease 1 [47]. Only polynucleotide phosphorylase 1 was detected in our sequencing, however, and the level of this protein was not changed after TBI (data not shown).

Recent studies suggest that miRNA levels are controlled by binding to other RNA molecules such as circularRNAs (circRNA), which can act as a sponge and inhibit the function of miRNAs [48]. Based on luciferase assays, miR-124 can bind to at least circHIPK3 [49], circMMP9 [50], circRNA_005186 [51], circRNA_100782 [52], and circWDR77 [53]. Among these, only circHIPK3 is expressed in the healthy brain [49]. Few studies have investigated the role of circRNA in TBI. Zhao et al. (2018) [54] sequenced exosomes derived from mouse brain after FPI and reported 231 differentially expressed circRNAs. The circRNAs that bind to miR-124-3p, however, were not among them. Similarly, sequencing of mouse cerebral cortex after controlled cortical impact revealed 191 differentially expressed circRNAs [55]. None of the levels of the validated miR-124-3p–targeting circRNAs were altered [55]. In another study on controlled cortical impact, sequencing of the peri-contusion area of the mouse cortex showed 16 differentially expressed circRNAs, none of them targeting miR-124-3p [56]. Taken together, the data linking circRNAs and miR-124-3p are insufficient and more studies are needed.

Finally, some studies suggest that supplementary binding to the target mRNA outside the seed region can alter the orientation of miRNA in the Ago protein [57], leading to tailing (addition of A/U non-template nucleotides), trimming, or shortening of the 3′-end, which results in miRNA degradation instead of mRNA silencing. This phenomenon, target RNA-directed microRNA degradation, is observed in *Drosophila* [58], zebra fish [59], mouse brain [59,60], and in cells infected by human cytomegalovirus [61,62,63]. For example, Bitetti et al. (2018) [59] showed that near-perfect binding between mouse Nrep (neuronal-regeneration-related protein) and miR-29b leads to 3′-trimming and decay of miR-29b in mouse cerebellum, triggering behavioral changes. Although evaluation of this phenomenon was outside the scope of the present study, we did notice that the binding of miR-124-3p varies between upregulated and downregulated targets, and that some upregulated targets had a complementary binding site outside the seed region. Further studies are needed to determine whether target RNA-directed microRNA degradation (TDMD) is the mechanism that underlies post-TBI miR-124-3p downregulation.

### 3.2. Downregulation of miR-124-3p Promotes Neurodegeneration

In our previous study, we showed that changes in miR-124-3p expression are comparable after experimental and human TBI as well as after status epilepticus-induced brain injury in rats [19]. Unexpectedly, we found that the sham-operation (i.e., craniotomy) also affects the miR-124-3p level, indicating that miR-124-3p could be highly responsive to skull and brain surgery. Interestingly, miR-124-3p is also downregulated in the cortex of mice with frontotemporal dementia [64], in the frontal cortex of patients with a behavioral variant of frontotemporal dementia [64], in patients with sporadic Alzheimer disease [65], and in cellular models of Parkinson disease [66] and Alzheimer disease [67]. Together, these findings indicate that miR-124-3p downregulation is broadly connected to brain injury and neurodegeneration, independent of the etiology.

Several studies have examined the link between miR-124-3p and apoptotic pathways. In a cell model of Parkinson disease, miR-124-3p overexpression promoted cell viability via suppression of the ANXA5/ERK signaling pathway [66], which was previously shown to promote apoptosis [68,69,70]. A study conducted on human SH-SY5Y cells showed that inhibiting miR-124-3p promoted apoptosis through the AMPK/mTOR pathway [71]. Moreover, miR-124-3p directly targets beta-secretase 1 [65,67], which has a crucial role in the formation of beta-amyloid, indicating that miR-124-3p downregulation contributes to the pathologic hallmarks of Alzheimer’s disease.

Interestingly, a recent in vitro study of apoptosis suggested that miR-124-3p could reprogram primary mouse astrocytes to exhibit a neuronal morphology and to express GABAergic or glutamatergic markers (conference abstract, [72]). This is consistent with the observation that miR-124-3p contributes to regulate neuronal identity [73]. Further, several studies indicate that targets of miR-124-3p promote cellular proliferation and migration in several cancers, including hepatocellular carcinoma [74,75], oral squamous cell carcinomas [76], and gastric cancer [77]. Moreover, in vitro studies demonstrated that transfection of premiR-124-3p in hepatocellular carcinoma cells induces apoptosis and limits proliferation [78,79]. Whether or not the apoptosis is triggered by pushing the non-neuronal cells toward a neuronal identity requires further clarification. The mixed effect of miR-124-3p depending on the target cell complicates the possible use of miR-124-3p as a systemically administered treatment and might require the development of neuron-specific delivery systems.

Our RT-qPCR analysis revealed a 7-fold upregulation of a target of miR-124, *Stat3*, in the perilesional cortex at 7 d post-TBI. We previously reported a similar post-TBI *Stat3* upregulation in the dentate gyrus [19]. Several different experimental models show post-injury activation of JAK2-STAT3 pathways [80,81,82,83,84]. Activation of the pathways occurs when JAK2 is phosphorylated. Inhibiting JAK2 and STAT3 phosphorylation leads to worsened neurologic recovery after TBI [85], while activating phosphorylation with human recombinant erythropoietin reduces post-TBI apoptosis of cortical cells in the rat brain [82]. Grabenstatter et al. (2014) [84] further demonstrated that brief inhibition of STAT3 phosphorylation reduces the frequency of spontaneous seizures in an animal model of temporal lobe epilepsy, suggesting that modulating *Stat3* levels through miR-124-3p could mechanistically contribute to post-traumatic epileptogenesis.

A striking number of studies connect miR-124-3p with neurologic diseases such as epilepsy, dementia, and Parkinson disease. This raises the question of whether miR-124-3p downregulation is a mechanism that contributes to the development of post-TBI comorbidities over a long period of time. In the present study, we analyzed patient samples obtained at a subacute post-TBI time-point. Our earlier study indicated that miR-124-3p can be downregulated, even for decades, after the initial brain insult [19]. Whether the downregulation of miR-124-3p expression initiates the apoptotic process at a given time-point or marks cells that have entered the apoptotic phase remains to be explored.

### 3.3. miR-124-3p Controls Anti-inflammatory Pathways

TBI leads to an inflammatory response, including activation of microglia and astrocytes, release of cytokines, and recruitment of peripheral immune cells such as macrophages [36]. Pathway analysis (e.g., IPA) of upregulated predicted targets of miR-124-3p indicated that miR-124-3p regulates inflammation via Toll-like receptor-mediated pathways. Lateral FPI leads to T-cell infiltration into perilesional cortex [86]. In addition to Toll-like receptor-pathways, miR-124-3p promotes the polarization of microglia towards anti-inflammatory M2 through the C/EBP-α pathway [87,88,89]. Further, miR-124-3p mediates the microglial inflammatory response via the MEKK/NF-κB signaling pathway [89,90]. Our in situ hybridization, double-labeled with cellular markers is in record with earlier studies, indicating that miR-124-3p is a neuronal miRNA [91,92]. Some studies have also suggested microglial expression of miR-124-3p [89]. However, neurons are known to actively secrete miR-124-3p to the extracellular space, after which miR-124-3p can be transferred to microglia, arguing against the intrinsic miR-124-3p expression in microglia [93]. Nevertheless, miR-124-3p downregulation is a potential chronic regulator of perilesional inflammation via its upregulated targets.

## 4. Materials and Methods

The study design is summarized in Figure 1.

### 4.1. Animals

Adult male Sprague-Dawley rats (Cohort 1: *n* = 25, body weight 381-425 g; Cohort 2: *n* = 23, body weight 332-391 g, Envigo, Udine, Italy; Cohort 3: *n* = 30, body weight 330–370 g at the time of injury, Harlan Laboratories S.r.l., Horst, Netherlands) were used. Rats were housed in a controlled environment (temperature 22 ± 1 °C; humidity 50–60%; lights on from 07:00 to 19:00 h). Water and pellet food were provided ad libitum. All procedures for rats with TBI were approved by the Animal Ethics Committee of the Provincial Government of Southern Finland. All animal work was carried out in accordance with the guidelines of the European Community Council Directives 2010/63/EU.

### 4.2. Lateral Fluid-Percussion-Induced TBI

Lateral fluid-percussion injury (FPI) was induced (Cohort 1: *n* = 15; Cohort 2: *n* = 12; and Cohort 3: *n* = 30, see: Figure 1) as described previously [94,95]. Briefly, animals were anesthetized by intraperitoneal injection (6 mL/kg) of a mixture of sodium pentobarbital (58 mg/kg), magnesium sulfate (127.2 mg/kg), propylene glycol (42.8%), and absolute ethanol (11.6%), and placed in a Kopf stereotactic frame (David Kopf Instruments, Tujunga, CA, USA). Chloral hydrate (60 mg/kg) was included in the anesthetic cocktail used in Cohort 3, but not in the anesthetic cocktail used in the other experiments due to changes in our animal license. A midline scalp incision was made, and the underlying periosteum dissected. A 5-mm circular craniectomy was performed with a hand-held trephine over the left parietal cortex, midway between lambda and bregma, with the lateral edge of the craniectomy adjacent to the lateral ridge. A modified Luer–lock cap was placed into the craniectomy, sealed with dental cement (Selectaplus CN, Dentsply DeTRey GmbH, Dreieich, Germany), and filled with saline. At 90 min after administration of the anesthesia, the rats were connected to the fluid-percussion device (AmScien Instruments, Richmond, VA, USA) through the male Luer–lock fitting and brain injury was induced (Cohort 1: 3.2 ± 0.02 atm; Cohort 2: 3.2 ± 0.02 atm; and Cohort 3: 3.3 ± 0.02 atm). Sham-operated experimental controls (Cohort 1: *n* = 10; Cohort 2: *n*= 8; and Cohort 3: *n* = 14) underwent all surgical procedures without lateral FPI. To investigate if the sham-operation affected miR-124 expression or its localization in the perilesional cortex, we also included naïve animals (*n* = 3) in Cohort 2. A subgroup of surviving animals was selected for analysis as summarized in Figure 1. In Cohort 1, six TBI and six sham animals were randomly selected for the analysis. In Cohort 2, one TBI and one sham animal were used for setting up the protocol, and were excluded from the final analysis. In the 3 months post-TBI group, one animal was excluded due to technical problems while preparing the brain. In Cohort 3, five TBI animals and five sham-treated controls were selected for sequencing based on the availability of tissue (at least 10 mg).

### 4.3. Detection of Cortical miR-124 Expression with RT-qPCR

Cortical expression of miR-124 was analyzed in Cohort 1.

#### 4.3.1. Tissue Sampling

At 7 d or 3 months post-TBI, animals were deeply anesthetized with isoflurane (5%) and decapitated with a guillotine. The brain was then removed from the skull, frozen in isopentane chilled with dry ice, and stored in −70 °C until further use.

#### 4.3.2. Laser Capture Microdissection (LCM)

Ten successive 10-µm-thick coronal sections containing the perilesional cortex (between 0.20 mm anterior and −4.52 mm posterior to bregma, rat brain atlas of Paxinos and Watson, (1986), [96]) were cut on LCM slides (#11505151, Leica, MicroDissect GmbH, Herborn, Germany) using a cryostat (Leica CM3050 S, Leica Microsystems Nussloch GmbH, Germany), and stored at −70 °C for a maximum of 48 h.

To recognize the cytoarchitectonic structures, the sections were stained with cresyl violet (Merck, Darmstadt, Germany). The sections were first fixed with ice-cold acetone for 15 min. The sections were hydrated by placing them in a graded series of ethanol (96% ethanol, 70% ethanol, and 50% ethanol; each for 30 s). They were then stained with 1% cresyl violet in 100% ethanol for 80 s. The sections were then dehydrated in a graded series of ethanol (50% ethanol, 70% ethanol, 70% ethanol, 96% ethanol, and 100% ethanol; each for 30 s). Finally, the sections were placed in xylene twice for 5 min each and dried under a hood for 10 min.

LCM was performed immediately after the cresyl violet staining. The perilesional cortex (aPeCx, see Figure 2A) and adjacent dorsally located somatosensory cortex (dPeCx, see Figure 2A) were outlined and cut with a Leica LMD Laser Microdissection System (Leica, Wetzlar, Germany) into QIAzol Lysis Reagent (Qiagen, Hilden, Germany, #79306: the tissue from all 10 sections was placed in one tube). To perform the immediate lysis reaction, the microdissected tissue in the lysis reagent was vortexed and centrifuged at room temperature for 2 min (800× *g*). Thereafter, the samples were stored at −70 °C until RNA extraction.

#### 4.3.3. RNA Extraction

RNA was extracted from the LCM samples using a miRNeasy Micro Kit (ID: 217084, Qiagen, Hilden, Germany) according to the manufacturer’s protocol. Briefly, total RNA was extracted using a centrifugation and chloroform-based phase separation method, and washed with 100% ethanol. For elution, RNA was bound to RNeasy MinElute spin columns and eluted with RNAse-free water. The RNA concentration and quality were measured with Nanodrop (ND-1000). Samples were stored at −70 °C until further processed.

#### 4.3.4. Reverse Transcription of RNA to Complementary DNA (cDNA) for microRNA Detection

Total RNA was translated to cDNA with the miScript PCR System (ID: 218193, Qiagen, Hilden, Germany) according to the manufacturer’s protocol. Briefly, 50 ng RNA was mixed with reverse transcription master mix (mixture of 5× miScript HiSpec Buffer, 10× miScript Nucleics Mix, miScript Reverse Transcriptase Mix, and nuclease free water). For reverse transcription, the samples were incubated for 60 min at 37 °C, followed by 5 min at 95 °C. The samples were stored at −20 °C until further use.

#### 4.3.5. Reverse Transcription of RNA to cDNA for mRNA Detection

To investigate if a changed miR-124-3p level affected its target expression, we investigated the expression of one of its most studied targets, *Stat3*. The interaction between miR-124-3p and *Stat3* has been previously verified by several groups [97,98,99]. For that, 76 ng of each sample was converted to cDNA using a High Capacity RNA-to-cDNA Kit (#4387406, Applied Biosystems, Foster City, CA, USA) according to the manufacturer’s protocol. In brief, RNA was mixed with 2× RT Buffer and 20× RT enzyme mix (Applied Biosystems, Foster City, CA, USA). RT reactions were carried out with the following program: 60 min at 37 °C, 5 min at 95 °C followed by cooling to +4°C. The samples were stored at −20 °C until further use.

#### 4.3.6. Quantitative PCR Analysis of Mature miRNAs

First, the primer for miR-124-3p (MS00005593, Qiagen, Hilden, Germany) was dissolved in Tris-EDTA (TE) buffer (10 nM Tris/HCl, 1 mM EDTA, pH 8.0), vortexed, and stored at −20 °C until used. The RT-qPCR was carried out as described in the miScript PCR System Handbook (https://www.qiagen.com/fi/resources/). Briefly, the samples were diluted by mixing 5 µL cDNA with 100 µL nuclease-free water. The PCR mixture (QuantiTect SYBR Green PCR Master Mix, miScript Universal Primer, miScript Primer Assay, RNase-free water, Qiagen, Hilden, Germany) was prepared as directed and mixed with 2.5 µL of the diluted sample on a PCR plate. The qPCR reaction was run in a Roche LightCycler 480 (Roche, Basel, Switzerland). The cycling conditions were as follows: to activate the PCR, the samples were incubated for 15 min at 95 °C. The samples then underwent 45 cycles of 15 s at 94 °C, 30 s at 55 °C, and 30 s at 70 °C. The miR-124 expression was normalized to that of a stable endogenous control, miR-378 (ID:218300, Qiagen, Hilden, Germany), using the 2^−ΔΔ*C*t^ method [100].

#### 4.3.7. Quantitative PCR Analysis of *Stat3*

The PCR mixture was prepared using 12 ng cDNA (RNA equivalents) as a template. The glyceraldehyde 3-phosphate dehydrogenase gene (Gapdh) was used as a stable endogenous control. Gene specific primers (prevalidated TaqMan Gene Expression Assay for *Stat3* ID; Rn01456553_m1, and Gapdh ID: Rn99999916_s1, Applied Biosystems, Foster City, CA, USA), TaqMan Gene expression Master Mix (#4399367, Applied Biosystems, Foster City, CA, USA), and the sample were mixed. Nuclease-free water was added to achieve a final volume of 20 µL per reaction. Quantitative PCR was run with the StepOnePlus real-Time PCR System (Applied Biosystems, Foster City, CA, USA) with the following program: 10 min at 95 °C, then 40 cycles including the first 15 s at 95 °C, and then 60 s at 60 °C. The relative *Stat3* expression was calculated using the 2^−ΔΔ*C*t^ method [100].

### 4.4. In Situ Hybridization of miR-124-3p in Rat

To investigate the perilesional expression and localization of miR-124 as well as the effect of cell loss on miR-124-3p expression, we conducted in situ hybridization in Cohort 2.

#### 4.4.1. Perfusion

At 7 d or 3 months post-TBI, the rats were anesthetized by intraperitoneal injection of pentobarbital (60 mg/kg) and transcardially perfused with 0.9% sodium chloride solution (30–35 mL/min for 2 min), followed by 4% paraformaldehyde in 0.1 M sodium phosphate buffer, pH 7.4 (5 mL/min for 20 min). The brain was removed from the skull and post-fixed in 4% paraformaldehyde in 0.1 M phosphate buffer for 4 h at 4 °C.

#### 4.4.2. Tissue Processing

First, the brains were rinsed in tap water for 30 min and placed in a Tissue-Tek Mega cassette (Sakura, #4173). Next, the brains were infiltrated with paraffin as follows (Shandon Citadel 2000): 1 h in 50% ethanol; 1 h in 80% ethanol; 1 h in 96% ethanol; twice for 2 h in 96% ethanol; and twice in paraffin (3 h and then 2 h). After embedding the brains in paraffin, they were stored at room temperature.

For in situ hybridization, the brains were cut into 6 µm-thick sections with a Microm 355 microtome and a Leica 818 blade. The sections were mounted on Superfrost microscope slides (Thermo scientific, Gebhard Menzel GmbH, Braunschweig, Germany) and stored at room temperature until further use. One TBI and one sham rat were used to validate the protocol and for double-staining.

#### 4.4.3. In Situ Hybridization

In situ hybridization was carried out as described earlier using 5′-3′fluorescein (FAM)-labeled probes (FAM-GgcAuuCacCgcGugCcuuA (capital letters indicate locked nucleic acids; lower case letters indicate 2′O-methylated nucleic acids), RiboTask APS, Odense, Denmark) [19]. Due to potential analysis-to-analysis variability in color development, tissues sampled from all animals at a given time-point were stained together.

#### 4.4.4. Assessment of the Intensity of miR-124-3p Expression in In Situ Hybridization

The RGB digital photographs of miR-124-3p in situ hybridization were obtained with an Axio Imager M2 microscope (Carl Zeiss Microimaging GmbH, Jena, Germany). All images were captured with identical exposure parameters using a 20× objective. ZEN 2 software (Carl Zeiss Microimaging GmbH, Jena, Germany) was used for image processing. The staining intensity was measured from digital photographs using ImageJ (v. 1.50i, [101,102]) software. The color threshold was adjusted to be comparable to the staining intensity of the original RGB photomicrograph. The images were then converted to grayscale. The region of interest (ROI) was outlined in the perilesional cortex (see Figure 1B), and the mean intensity of the hybridization signal within the ROI was calculated. To strengthen the analysis, we also measured the intensity of the in situ hybridization signal in each individual cell inside the ROIs. The intensity of the hybridization signal was calculated using the following equation: (mean intensity of background ROI—mean intensity of the ROI)/mean intensity of background ROI. Background staining (background ROI) was measured from the ipsilateral fimbria.

#### 4.4.5. Double-Labeling with Cellular Markers

In situ hybridization was performed on a subset of 7-d post-TBI sections as described above. After in situ hybridization, the sections were washed three times with phosphate-buffered saline (PBS) and incubated with primary antibody against glial fibrillary acidic protein (astrocytes, 1:2000, G-3893, Clone G-A-5, Sigma-Aldrich, Munich, Germany), Iba1 (microglia, 1:200, 019-19741, WAKO, Tokyo, Japan), or NeuN (neuronal nuclei, 1:2000, MAB377, clone A60, Chemicon, Temecula, CA, USA) in room temperature for 60 min. The sections used for NeuN detection were then washed three times with PBS and blocked with post-antibody blocking solution (BrightVision plus Kit, Immunologic, Duiven, The Netherlands) at room temperature for 15 min. All sections were then washed three times with PBS. Poly-HRP-GAMs/Rb IgG was added to the sections and the sections were incubated at room temperature for 30 min. To detect the peroxidase activity, sections were incubated with aminoethylcarbazole (in 0.05 M saline buffer, pH 4.9 with 0.01% H_2_O_2_) for 8 min in the dark. To stop the reaction, the sections were first washed in deionized water then in tap water. The sections were dehydrated by dipping them into 70% ethanol for 1 min, twice into absolute ethanol for 1 min, and three times into xylene for 1 min. Finally, the sections were coverslipped using glycerol gelatin.

### 4.5. In situ Hybridization of miR-124-3p in Human TBI Brain

Expression of miR-124 after TBI was further studied in samples from the perilesional cortex of TBI patients.

#### 4.5.1. Sampling

Autopsy tissue from patients with TBI was available from the Department of Neuropathology of the Academic Medical Center in Amsterdam (Table 1). We examined a total of six specimens (5 men, 1 woman, median age 73 years, range 20–82 years). Control samples were obtained at the autopsy from three adult patients (3 men, mean age 60 y, range 20–82 years) without a history of neurologic disease. All samples were collected within 24 h after death. Informed consent was obtained for the use of the brain tissue and for access to the patients’ medical records. The tissue was obtained and used in accordance with the Declaration of Helsinki and the Academic Medical Center Research Code provided by the Medical Ethics Committee.

#### 4.5.2. Tissue Processing

Post-mortem tissue samples were fixed in 10% buffered formalin and embedded in paraffin. Tissue blocks were sectioned with a microtome at 6 µm and mounted on precoated glass slides (Star Frost, Waldemar Knittel, Braunschweig, Germany).

#### 4.5.3. In Situ Hybridization

In situ hybridization was performed as described above for the rat tissue except that the probe concentration was 250 nM.

#### 4.5.4. Intensity Analysis

Our initial analysis indicated that the TBI patients had a patchy neuronal loss in layer III, which, in the control cases, was densely packed with miR-124–positive cells. Consequently, we selected one representative case to measure the intensity of the in situ hybridization signal in individual cortical cells within a 100 µm-wide sector inside and outside of the patchy area.

### 4.6. Bioinformatic Analysis of miR-124 Targets

To investigate whether lateral FPI affects miR-124–controlled molecular pathways in the perilesional cortex, we used the RNA-sequencing data from Cohort 3. Tissue sampling, a detailed protocol for RNA-sequencing and data analysis were reported earlier [103]. The perilesional cortex was dissected between −2.2 to −6.2 from bregma as illustrated in Figure 1C.

TargetScan (version 7.2; [104]) was used to retrieve the targets of miR-124-3p that are expressed in rats and have conserved binding sites for miR-124-3p in their 3′UTR region. Altogether, we found 1547 transcripts with a conserved target site for miR-124-3p. Targets with a significant (*p* < 0.05 as compared to sham-operated controls) expression change after lateral FPI (312 upregulated, 311 downregulated targets of miR-124) were further analyzed. The data were investigated with a gene set enrichment analysis (GSEA, [105,106]. A preranked list was created from the RNA-sequencing dataset on the basis of the fold-change and *p*-values, as described previously [19]. To investigate the function of the altered miR-124 interactome, regulated targets were entered into the Reactome Pathway Browser (v3.9, [107] Fabregat et al., 2016, database updated in June 2019).

To further investigate the connection between miR-124 targets and known disease-related pathways, we entered the list of regulated targets into the ingenuity pathway analysis (IPA v.01-12, Qiagen, http://www.qiagen.com/ingenuity, [108]). We included into the analysis only experimentally observed interactions in the rat or human nervous system.

To investigate the miRNA-target binding properties, we used RNAhybrid [109] and TargetScan to investigate the top 10 downregulated and top 10 upregulated targets (based on GSEA; http://software.broadinstitute.org/gsea).

### 4.7. Statistical Analysis

Statistical analyses were performed using IBM SPSS Statistics 25.0. (IBM Corp., Armonk, NY, USA). The data were analyzed using the non-parametric Kruskal–Wallis ANOVA test followed by post hoc analysis with the Mann–Whitney U test. A *p*-value less than 0.05 was considered statistically significant. For detection of possible outliers, we used the Grubb’s test using an online calculator by GraphPad (GraphPad Software, San Diego, California, USA. Available at https://www.graphpad.com/quickcalcs/grubbs1/).

## 5. Conclusions

TBI induces a chronic downregulation of miR-124-3p in the perilesional cortex. Unexpectedly, sham-operation also slightly downregulated the cortical levels of miR-124-3p, indicating that even just craniectomy under isoflurane anesthesia, which is often used as a control procedure, can induce tissue pathology. In silico analysis indicated that the differentially expressed miR-124-3p targets have variable miR-124-3p binding efficacy. Regarding the recent findings on miRNA stability and regulation in the brain, the variability in binding efficiency could play a role in miR-124-3p downregulation. Findings of miR-124-3p downregulation also in the cortex of TBI patients indicate the translatability of the experimental findings to the clinic. Further studies are warranted to explore the potential miR-124-3p as a possible target intervention after TBI.

## Figures and Tables

**Table 1:** Patient information.

Sex	Post-TBI	Age at the Time of Death	Injury Mechanism	Brain Region	Injury Severity (GCS)	Cause of Death	Epilepsy (yes/no)	CT
m	5 days	20	Car accident	TC HC+TC	Severe	Trauma	no	Cerebral edema, small hemorrhages, diffuse axonal injury
m	8 days	79	Fall accident	BA38	Moderate	Post-traumatic pneumonia	no	Cerebral edema
m	2 months	82	Fall accident	BA21 HC+TC	Mild	Cardiorespiratory insufficiency	no	N/A
f	N/A	64	N/A	N/A	N/A	Myocardial infarction	no	N/A
m	N/A	67	N/A	N/A	N/A	Cardiorespiratory insufficiency	no	N/A
m	N/A	81	N/A	N/A	N/A	Myocardial infarction	no	N/A

Mild head injury (GCS score of 13–15), moderate head injury (GCS score of 9–12), severe head injury (GSC score 3–8). TC—temporal cortex, HC—hippocampus, BA—Brodmann area, m—male, f—female, N/A—not applicable.

**Table 2:** The Reactome analysis linked the TBI-regulated targets of miR-124-3p to signal transduction and immunity. The 20 pathways with the most mapped targets are presented.

Upregulated Targets of miR-124-3p			Downregulated Targets of miR-124-3p		
Pathway	Subpathway	Molecules	Pathway	Subpathway	Molecules
S	Signaling by Receptor Tyrosine Kinases	25	NS	Neuronal System	30
S	Signaling by Nuclear Receptors	16	DB	Axon guidance	21
S	ESR-mediated signaling	13	NS	Transmission across Chemical Synapses	18
MP	IRE1alpha activates chaperones	9	MC	Muscle contraction	14
MP	Unfolded Protein Response (UPR)	9	MC	Cardiac conduction	13
MP	XBP1(S) activates chaperone genes	8	NS	Neurotransmitter receptors and postsynaptic signal transmission	12
DB	Semaphorin interactions	7	NS	Potassium Channels	8
DB	Transcriptional regulation of granulopoiesis	7	NS	Protein-protein interactions at synapses	7
S	Extra-nuclear estrogen signaling	7	S	Opioid Signaling	7
S	Signaling by NTRKs	7	GE	MECP2 regulates neuronal receptors and channels	6
TSM	Plasma lipoprotein assembly, remodeling, and clearance	6	MC	Phase 0—rapid depolarization	6
M	Import of palmitoyl-CoA into the mitochondrial matrix	5	M	Regulation of cholesterol biosynthesis by SREBP (SREBF)	6
CD	Apoptotic execution phase	5	IS	MyD88 cascade initiated on plasma membrane	6
D	Diseases associated with glycosaminoglycan metabolism	5	IS	Toll Like Receptor 5 (TLR5) Cascade	6
NS	GABA receptor activation	5	IS	Toll Like Receptor 10 [TLR10] Cascade	6
S	NRAGE signals death through JNK	5	GE	Transcriptional Regulation by MECP2	6
H	Basigin interactions	4	IS	TRAF6 mediated induction of NFkB and MAP kinases upon TLR7/8 or 9 activation	6
EMO	Laminin interactions	4	IS	MyD88 dependent cascade initiated on endosome	6
GE	TP53 Regulates Transcription of Genes Involved in Cytochrome C Release	4	IS	Toll Like Receptor 7/8 (TLR7/8) Cascade	6
CD	Apoptotic cleavage of cellular proteins	4	IS	Toll Like Receptor 9 (TLR9) Cascade	6

CD, cell death; D, disease; DB, developmental biology; EMO, extracellular matrix organization; GE, gene expression; H, hemostasis; IS, immune system; M, metabolism, MC; muscle contraction; MP, metabolism of proteins; NS, neuronal system; S, signaling; TSM, transport of small molecules.

**Table 3:** Ingenuity pathway analysis of miR-124-3p targets with differential expression in the perilesional cortex at 3 months post-TBI. Twenty pathways with the most mapped targets are presented.

Upregulated Targets of miR-124-3p			Downregulated Targets of miR-124-3p		
Category	Diseases or Functions Annotation	Molecules	Category	Diseases or Functions Annotation	Molecules
NSD	Cell death of cortical neurons	14	CM	Microtubule dynamics	18
CDN	Collapse of growth cone	5	NSD	Development of neurons	17
CDN	Neuroprotection of cerebral cortex cells	4	NSD	Neuritogenesis	14
NSD	Cell viability of hippocampal neurons	3	NSD	Proliferation of neuronal cells	11
CDN	Growth of embryo	3	CDN	Necrosis	11
NSD	Neurite outgrowth stage of dorsal root ganglion cells	3	CDN	Cell death of brain cells	10
NSD	Neuroprotection of hippocampus	2	NSD	Growth of neurites	10
NSD	Differentiation of embryonic cells	2	CDN	Neurological signs	9
NSD	Hereditary neuropathy	2	CDN	Alzheimer disease	9
CDN	Invasion of endothelial cells	2	CDN	Cell death of cerebral cortex cells	8
CDN	Development of enteric nervous system	1	NSD	Branching of neurites	8
NSD	Survival of neural tube cells	1	CDN	Huntington Disease	8
NSD	Prostatic carcinoma	1	NSD	Dendritic growth/branching	7
CDN	Apoptosis of neural tube cells	1	NSD	Plasticity of synapse	5
CDN	Inhibition of sympathetic nerve	1	NSD	Density of neurons	5
NSD	Apoptosis of pericytes	1	CC	Cell-cell contact	5
CDN	Quantity of long-chain acyl-coenzyme A	1	NSD	Quantity of neurons	5
M	Proliferation of neural tube cells	1	CDN	Cell death of cortical neurons	5
NSD	Finnish type amyloidosis	1	CC	Synaptic depression	4
CDN	Cell death of cortical neurons	1	NSD	Developmental process of synapse	4

CC, cell-to-cell -contact; CDN: cell death and neurodegeneration; M, metabolism; NSD, nervous system development.

## Supplementary Materials

Supplementary materials can be found at https://www.mdpi.com/1422-0067/21/7/2418/s1.

## Abbreviations

*Abhd4*	abhydrolase domain containing 4
aPeCx	perilesional cortex adjacent to the lesion core
*Atmin*	ATM interactor
*Atp1a1*	ATPase Na+/K+ transporting subunit alpha 1
BA	Brodmann area
*Bcat1*	branched chain amino acid transaminase 1
*Cadps*	calcium dependent secretion activator;
circRNA	circularRNA
*Cnn3*	calponin 3
d	day
*Dnase2*	deoxyribonuclease 2
dPeCx	somatosensory cortex distal to the lesion
ES	enrichment score
FC	fold change
FDR	false discovery rate
FPI	fluid-percussion injury
GSEA	Gene Set Enrichment Analysis
*Gsn*	gelsolin
HC	hippocampus
*Hivep2*	HIVEP zinc finger 2
LCM	lacer capture microscopy
*Lcp1*	lymphocyte cytosolic protein 1
*Litaf*	lipopolysaccharide-induced TNF factor
*Mark1*	microtubule affinity regulating kinase 1
mo	month
*Nat8l*	N-acetyltransferase 8-like
*Nptn*	neuroplastin
*Nrep*	neuronal-regeneration-related protein
*Osbp2*	oxysterol binding protein 2
ROI	region of interest
RT-qPCR	quantitative reverse transcriptase polymerase chain reaction
*Scamp2*	secretory carrier membrane protein 2
*Sdc4*	syndecan 4
*Stat3*	Signal transducer and activator of transcription 3
TBI	traumatic brain injury
TC	temporal cortex
TDMD	target RNA-directed microRNA degradation
*Ttl*	tubulin tyrosine ligase
UTR	untranslated region
*Vim*	vimentin
*Zfp36l1*	zinc finger protein 36 C3H type-like 1
