# Supplementary material for: Chronic Regulation of miR-124-3p in the Perilesional Cortex after Experimental and Human TBI
Source: Int J Mol Sci. 2020 Mar 31;21(7):2418. doi: 10.3390/ijms21072418 (PMC7177327; doi:10.3390/ijms21072418)
Supplement: Supplementary file 1 [file ijms-21-02418-s001.pdf]

Supplementary Materials

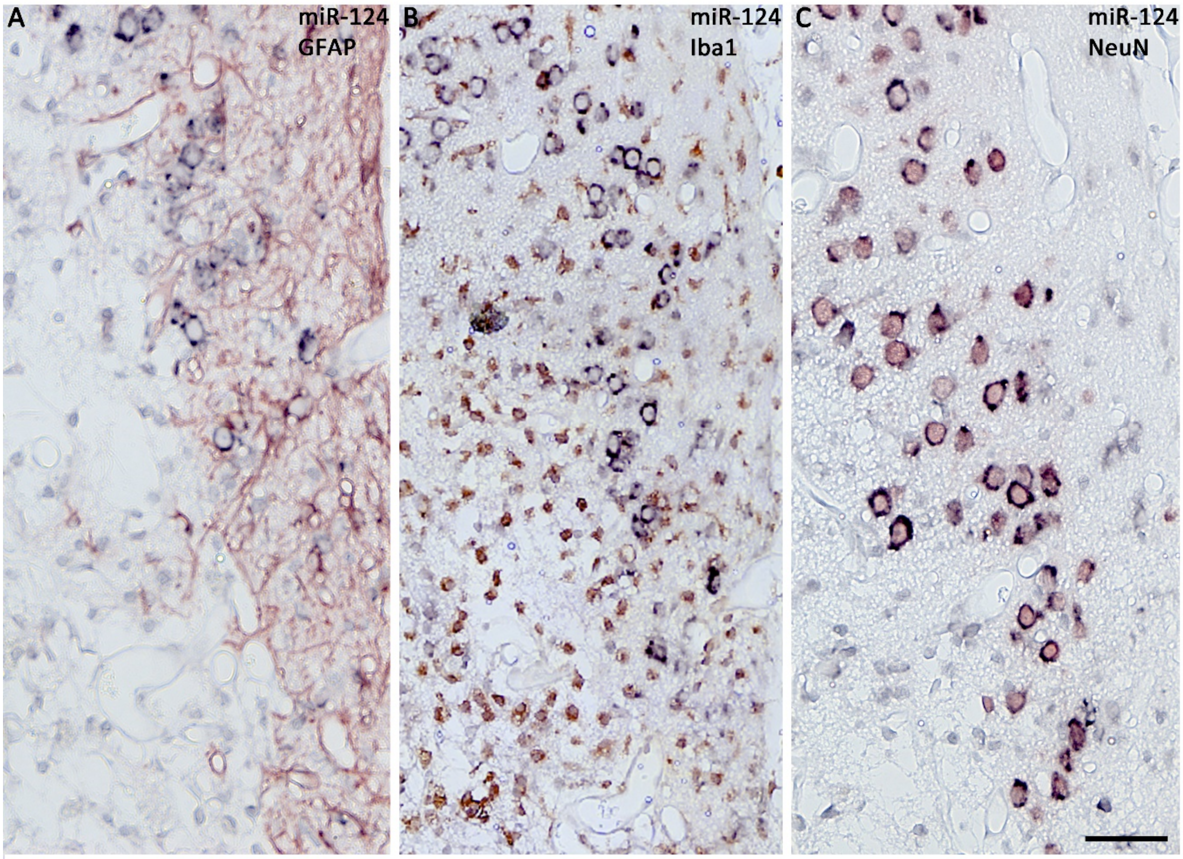

Supplementary Figure S1. Post-TBI localization of miR-124-3p in rat perilesional cortex. *In situ* hybridization of miR-124-3p and double-labeling with glial fibrillary acidic protein (GFAP; **A**, astrocytes), Iba1 (**B**, microglia), and NeuN (C, neurons) shows that miR-124-3p was expressed exclusively in neurons. Scale bar 50 µm.

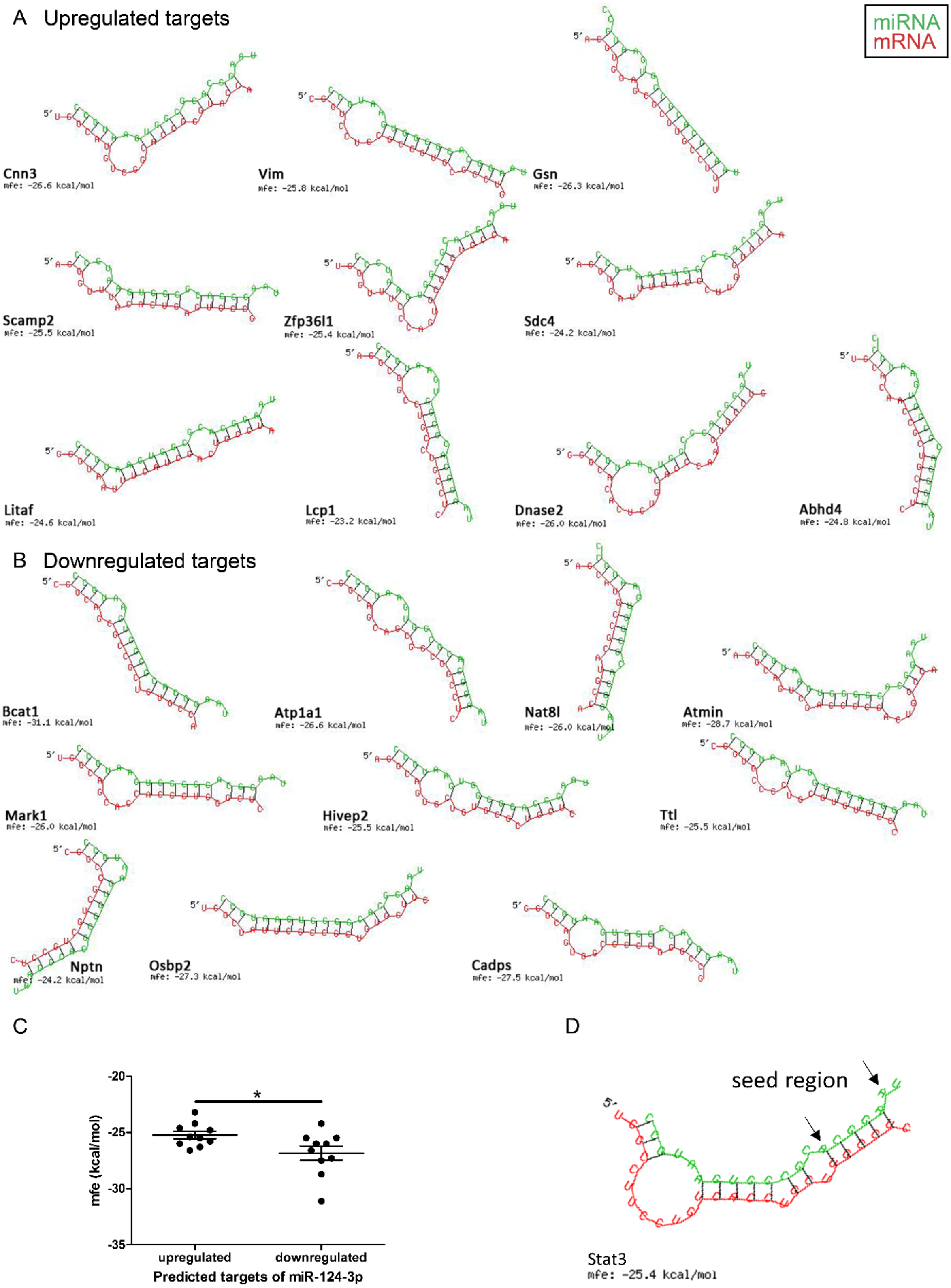

Supplementary Figure S2. Binding of miR-124-3p to the top 10 upregulated and downregulated targets on the basis of Gene Set Enrichment Analysis. RNAhybrid analysis on the top 10 upregulated (**A**) and downregulated (**B**) targets of miR-124-3p revealed that the downregulated targets had a lower minimum free energy compared with upregulated targets (**C**, $p < 0.05$), indicating stronger bonds between these targets and miR-124-3p. Interestingly, the analysis indicated that in all investigated targets, miRNA bound to the target from nucleotides 3-4. (**D**) This was also the case with Stat3.

Abbreviations: Abhd4, abhydrolase domain containing 4; Atmin, ATM interactor; Atp1a1, ATPase Na+/K+ transporting subunit alpha 1; Bcat1, branched chain amino acid transaminase 1; Cadps, calcium dependent secretion activator; Cnn3, calponin 3; Dnase2, deoxyribonuclease 2; Gsn, gelsolin; Hivep2, HIVEP zinc finger 2; Lcp1, lymphocyte cytosolic protein 1; Litaf, lipopolysaccharide-induced TNF factor; Mark1, microtubule affinity regulating kinase 1; Nat8l, N-acetyltransferase 8-like; Nptn, neuroplastin; Osbp2, oxysterol binding protein 2; Scamp2, secretory carrier membrane protein 2; Sdc4, syndecan 4; Stat3, Signal transducer and activator of transcription 3; Ttl, tubulin tyrosine ligase; Vim, vimentin; Zfp36l1, zinc finger protein 36, C3H type-like 1. Statistical significance: *<0.05 (Mann-Whitney U).

Supplementary Table S1. Proteins linked with processing of pre-miRNA to mature miRNA [44] and their expression in perilesional cortex at 3 months post-TBI.

**Gene name**	**Gene annotation**	***p*-value**	**log2FoldChange**
ADAR	adenosine deaminase, RNA-specific	0.276	−0.115
AGO1	argonaute RISC component 1	0.415	−0.110
AGO2	Argonaute RISC catalytic component 2	0.070	−0.264
AGO3	Argonaute RISC catalytic component 3	0.134	−0.285
AGO4	Argonaute RISC component 4	0.109	−0.251
BCDIN3D	BCDIN3 domain containing RNA methyltransferase	0.137	−0.173
DICER1	Dicer 1 ribonuclease III	0.608	−0.040
DROSHA	Drosha ribonuclease III	0.166	−0.089
LIN28A	Lin-28 homolog A	0.250	0.322
LIN28B	Lin-28 homolog B	0.180	−0.380
MRPL44	Mitochondrial ribosomal protein L44	0.024	−0.177
PRKRA	Protein activator of interferon induced protein kinase EIF2AK2	0.265	0.113
TARBP2	TARBP2 subunit of RISC loading complex	0.140	−0.167
TUT4	terminal uridylyl transferase 4	NA	NA
TUT7	Terminal uridylyl transferase 7	NA	NA

[44] L. He and G. J. Hannon, "MicroRNAs: small RNAs with a big role in gene regulation," *Nat. Rev. Genet.*, vol. 5, no. 7, pp. 522–531, Jul. 2004, doi: 10.1038/nrg1379.
